# Causal spectral segmenter: counterfactual graph reasoning for weakly supervised pathology segmentation

**DOI:** 10.1093/bioinformatics/btag674

**Published:** 2026-09-10

**Authors:** Xu Zhang, Jiasheng Si, Wenpeng Lu, Cheng Li, Xinbin Zhang

**Affiliations:** Shandong Cancer Hospital and Institute, Shandong First Medical University and Shandong Academy of Medical Sciences, Jinan, China; Key Laboratory of Computing Power Network and Information Security, Ministry of Education, Shandong Computer Science Center (National Supercomputer Center in Jinan), Qilu University of Technology (Shandong Academy of Sciences), Jinan, China; Shandong Provincial Key Laboratory of Computing Power Internet and Service Computing, Shandong Fundamental Research Center for Computer Science, Jinan, China; Key Laboratory of Computing Power Network and Information Security, Ministry of Education, Shandong Computer Science Center (National Supercomputer Center in Jinan), Qilu University of Technology (Shandong Academy of Sciences), Jinan, China; Shandong Provincial Key Laboratory of Computing Power Internet and Service Computing, Shandong Fundamental Research Center for Computer Science, Jinan, China; Shandong Cancer Hospital and Institute, Shandong First Medical University and Shandong Academy of Medical Sciences, Jinan, China; Shandong Cancer Hospital and Institute, Shandong First Medical University and Shandong Academy of Medical Sciences, Jinan, China

## Abstract

**Motivation:**

Weakly supervised pathology segmentation aims to alleviate the reliance on costly pixel-level annotations, yet remains challenging due to severe tissue heterogeneity, complex morphological patterns, and strong contextual confounding in histopathology images. Existing transformer-based methods often learn spurious correlations between lesions and surrounding tissues, while graph-based approaches tend to suffer from feature over-smoothing, resulting in degraded boundary delineation and localization accuracy.

**Results:**

To address these challenges, we propose a novel *Causal Spectral Segmenter (CSS)* for weakly supervised pathology segmentation. The proposed framework seamlessly integrates causal representation learning and multi-scale spectral graph reasoning. Specifically, a *Causal Counterfactual Projector (CCP)* is introduced to estimate lesion-specific causal effects through factual–counterfactual intervention, thereby suppressing contextual confounding and enhancing lesion-discriminative representations. Furthermore, we develop a *Multi-scale Spectral Graph Reasoner (MSGR)* composed of stacked *Spectral Chebyshev Graph Convolution (SCGC)* layers, which perform topology-aware spectral propagation across multiple neighborhood scales to capture long-range tissue dependencies while mitigating graph over-smoothing. By jointly modeling causal effects and multi-scale topological structures, CSS effectively improves lesion localization and boundary preservation under weak supervision. Extensive experiments on two public histopathology segmentation benchmarks demonstrate that CSS consistently outperforms state-of-the-art methods and achieves superior segmentation accuracy and structural consistency.

**Availability and implementation:**

The source code and implementation details will be publicly available at: https://github.com/zhangxu90s/CSS.

## 1 Introduction

Pathology image segmentation is a fundamental task in computational pathology, playing a crucial role in automated lesion localization, disease grading, quantitative tissue analysis, and downstream clinical decision support (Campanella *et al.* 2019, Chikontwe *et al.* 2022). Accurate segmentation enables the delineation of tumor regions and microenvironmental structures, facilitating the assessment of disease progression and treatment response. With the rapid digitization of whole-slide imaging, pathology segmentation has become an indispensable component of modern computer-aided diagnosis systems.

Despite the success of deep learning in medical image analysis, pathology segmentation remains challenging due to the high intra-class variability and inter-class similarity of histopathology images. Tissue structures such as tumor epithelium, stroma, necrosis, and lymphocytes exhibit complex morphology, ambiguous boundaries, and heterogeneous spatial distributions. In addition, staining variations, scanner differences, and specimen preparation further increase inter-domain variability, making robust lesion representation learning difficult and limiting generalization. To reduce reliance on pixel-level annotations, weakly supervised pathology segmentation has gained increasing attention (Lu *et al.* 2021, Han *et al.* 2022, Li *et al.* 2025). However, weak supervision inevitably introduces ambiguity, causing models to rely on spurious contextual correlations rather than true pathological evidence. As a result, lesion features are often entangled with surrounding tissue context, leading to unstable and unreliable segmentation performance.

Recent transformer-based architectures have demonstrated strong capability in modeling long-range dependencies through global self-attention mechanisms (Li *et al.* 2023, Gao *et al.* 2025). By aggregating information from distant tissue regions, these methods provide powerful contextual representations for pathology understanding. However, the global aggregation nature of self-attention may unintentionally amplify contextual bias, making lesion-specific evidence increasingly entangled with background information. Meanwhile, graph neural networks offer a complementary paradigm by explicitly modeling structural relationships among image patches (Zhang *et al.* 2022, Ngo *et al.* 2025). Nevertheless, most existing graph-based approaches rely on isotropic neighborhood aggregation, which often results in progressive feature over-smoothing as graph propagation depth increases. Such over-smoothing weakens discriminative boundary information and limits the ability of graph models to accurately delineate complex lesion structures.

Motivated by these observations, we argue that an effective weakly supervised pathology segmentation framework should adopt a causal perspective to mitigate spurious contextual correlations (Si *et al.* 2024, Zhang *et al.* 2024, Li *et al.* 2026), and satisfy two essential requirements: (1) explicitly suppress contextual confounding and enhance lesion-specific representations, and (2) preserve discriminative structural details while modeling long-range tissue dependencies.

Specifically, we introduce a *Causal Counterfactual Projector (CCP)* that estimates lesion-specific causal effects through factual–counterfactual intervention, enabling the model to disentangle lesion-relevant evidence from contextual confounders. Furthermore, we develop a *Multi-scale Spectral Graph Reasoner (MSGR)* composed of stacked *Spectral Chebyshev Graph Convolution (SCGC)* layers. The proposed MSGR performs topology-aware spectral propagation across multiple neighborhood scales, allowing adaptive frequency modeling while alleviating graph over-smoothing and preserving fine-grained lesion boundaries. By jointly modeling causal effects and multi-scale topological structures, CSS effectively improves lesion localization and structural consistency under weak supervision.

Extensive experiments on public weakly supervised pathology segmentation benchmarks demonstrate that the proposed framework consistently outperforms existing state-of-the-art methods, validating the effectiveness of integrating causal counterfactual learning with spectral graph reasoning.

The main contributions of this work are summarized as follows:

- We propose a novel *Causal Counterfactual Projector (CCP)* that estimates lesion-specific causal effects through factual–counterfactual intervention, effectively mitigating contextual confounding in weakly supervised pathology segmentation.
- We introduce a *Multi-scale Spectral Graph Reasoner (MSGR)* based on stacked *Spectral Chebyshev Graph Convolution (SCGC)* layers, which performs multi-scale frequency-aware topology reasoning and alleviates graph over-smoothing while preserving fine-grained lesion boundaries.
- We develop a unified *Causal Spectral Segmenter (CSS)* that seamlessly integrates causal representation learning and spectral graph reasoning, achieving superior performance on challenging weakly supervised pathology segmentation benchmarks.

## 2 Method

**Figure 1:**
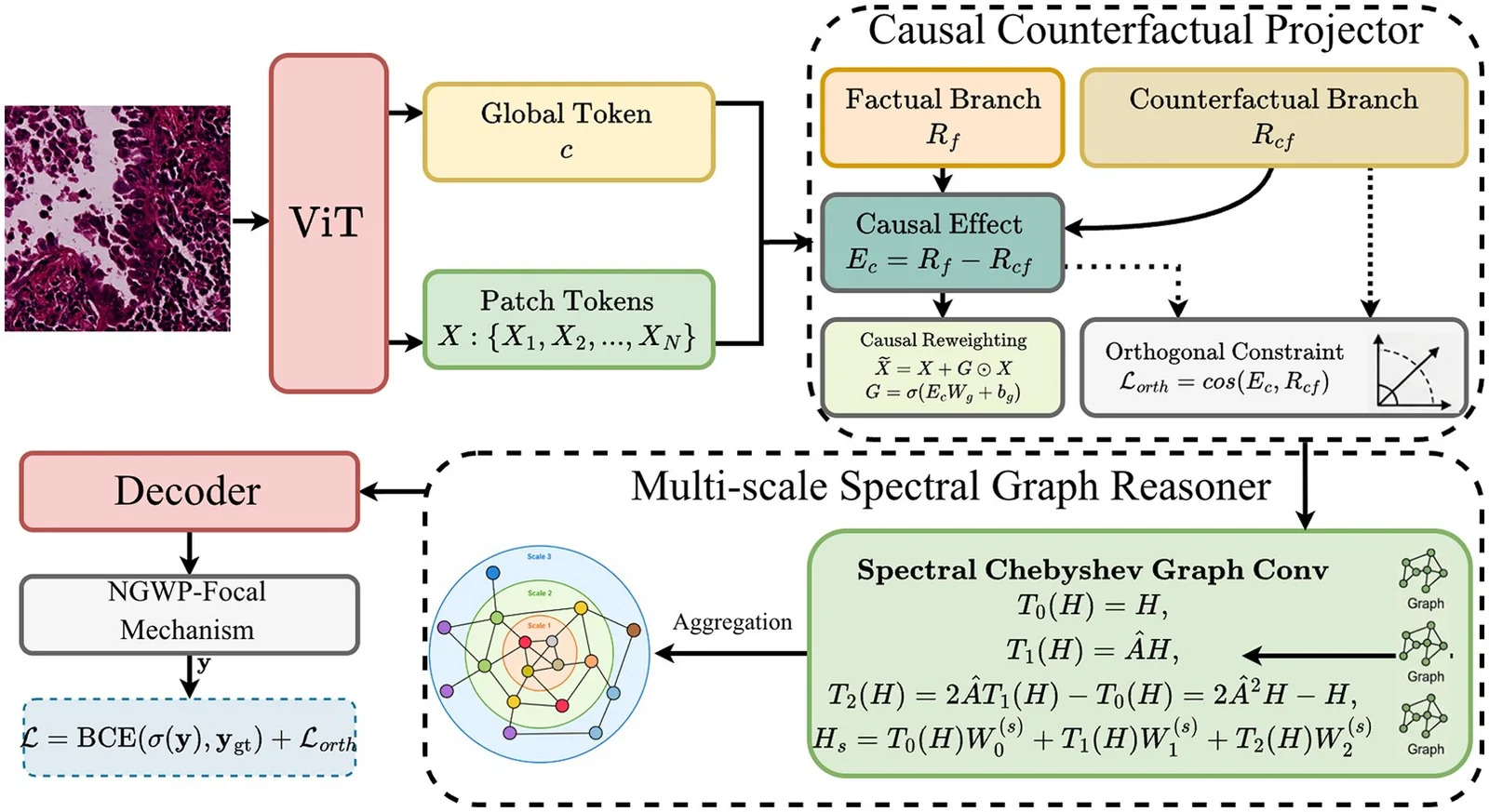
The architecture of CSS.

Weakly supervised pathology segmentation aims to localize lesion regions using only coarse annotations such as image-level labels while avoiding the expensive acquisition of dense pixel-level masks. Despite recent advances in transformer-based and graph-based architectures, accurate lesion localization remains challenging due to two fundamental issues. First, pathology images often exhibit strong contextual confounding caused by tissue heterogeneity, staining variations, and slide-specific acquisition conditions, leading models to exploit spurious contextual correlations instead of genuine pathological evidence. Second, although graph reasoning can effectively capture structural dependencies among tissue regions, repeated neighborhood aggregation often introduces feature over-smoothing, which weakens discriminative boundaries and degrades fine-grained lesion delineation. To address these challenges, we propose a novel *Causal Spectral Segmenter (CSS)*, which seamlessly integrates causal representation learning and multi-scale spectral graph reasoning within a unified weakly supervised segmentation framework. As illustrated in Fig. 1, CSS consists of three major components:

- A Vision Transformer (ViT) encoder that extracts global and local pathology representations from image patches;
- A *Causal Counterfactual Projector (CCP)* that estimates lesion-specific causal effects through factual–counterfactual intervention and suppresses contextual confounding;
- A *Multi-scale Spectral Graph Reasoner (MSGR)* composed of stacked *Spectral Chebyshev Graph Convolution (SCGC)* layers, which perform topology-aware spectral propagation across multiple neighborhood scales while preserving lesion boundaries.

Given an input pathology image $I\in R^{H\times W\times 3}$, the ViT encoder first generates a global classification token together with a sequence of patch-level embeddings. The CCP module then performs causal intervention to disentangle lesion-related information from contextual bias and produces causally refined patch representations. Subsequently, the refined tokens are projected onto a collection of multi-scale topology graphs and propagated through the MSGR module for structural reasoning. Finally, a transformer decoder generates dense segmentation predictions from the graph-enhanced representations.

### 2.1 Causal counterfactual projector

#### 2.1.1 Causal motivation and structural causal model

A fundamental challenge in weakly supervised pathology segmentation arises from the discrepancy between image-level supervision and pixel-level localization. Since optimization is driven only by global image labels, the model is encouraged to discover any predictive signal correlated with the target category, regardless of whether such signals correspond to true lesion evidence.

Let *Z* denote latent lesion-specific pathological characteristics, such as tumor morphology, cellular arrangement, and discriminative tissue structures. Let *C* represent contextual factors, including surrounding tissue composition, stromal background, staining style, scanner-dependent appearance, and slide-specific acquisition variations. Under weak supervision, the learned visual representation is typically an entangled mixture of *Z* and *C*. Consequently, segmentation predictions may rely on spurious contextual cues rather than genuine lesion structures.

To explicitly clarify the causal assumptions of the proposed CCP, we formulate weakly supervised pathology segmentation using a structural causal model (SCM). Let I denote the observed pathology image, X denote the patch-token representation extracted by the encoder, R denote the attention response used for prediction, and $\hat{Y}$ denote the predicted segmentation map. The structural equations are written as


(1) $$Z=f_{Z}(U_{Z}),\;C=f_{C}(U_{C}),$$

(2) $$I=f_{I}(Z,C,U_{I}),$$

(3) $$X=f_{\phi }(I),$$

(4) $$R=f_{A}(X,C;\theta ),$$

(5) $$\hat{Y}=f_{\psi }(X,R;\theta ),$$

where $U_{Z}$, $U_{C}$, and $U_{I}$ are exogenous noise variables, $f_{\phi }$ denotes the encoder, $f_{A}$ denotes the attention-based response function, and $f_{\psi }$ denotes the segmentation decoder.

Ideally, the ground-truth segmentation should be mainly determined by the lesion-specific factor *Z*. However, because image-level supervision does not provide dense spatial constraints, the learned prediction $\hat{Y}$ may depend on both *Z* and contextual factor *C*. Therefore, the desired lesion-specific causal pathway can be represented as


(6) $$Z\to I\to X\to R\to \hat{Y},$$

whereas the spurious context-dependent pathway can be represented as


(7) $$C\to I\to X\to R\to \hat{Y}.$$

This causal view explains why weakly supervised models may incorrectly activate surrounding stromal tissue, necrotic background, or other visually correlated non-target regions. These contextual regions may be statistically associated with the image-level label in the training set, but they are not necessarily causal evidence for the target tissue category. Such contextual dependency becomes particularly problematic when the contextual distribution changes across slides, staining protocols, scanners, or medical centers.

The goal of the proposed Causal Counterfactual Projector (CCP) is therefore to construct a representation-level counterfactual intervention that suppresses the context-dominated pathway while preserving lesion-specific evidence. Specifically, CCP compares the factual attention response with a counterfactual response in which local value tokens are replaced by a homogeneous contextual prototype. The resulting factual–counterfactual contrast estimates lesion-specific causal effects and reduces reliance on irrelevant contextual information.

#### 2.1.2 Factual–counterfactual intervention

Let


(8) $$X=[x_{1},x_{2},\ldots ,x_{N}]\in R^{N\times d}$$

denote the patch-token sequence extracted by the ViT encoder, where *N* is the number of patches and *d* is the embedding dimension. Let


(9) $$c\in R^{d}$$

represent the global classification token.

The classification token is projected into a query representation,


(10) $$q=W_{q}c,$$

while patch tokens are transformed into key and value embeddings:


(11) $$K=W_{k}X,\;V=W_{v}X,$$

where $W_{q}$, $W_{k}$, and $W_{v}$ are learnable projection matrices. The attention weight matrix is computed as


(12) $$A_{\mathrm{att}}=\text{Softmax}\left(\frac{qK^{⊤}}{\sqrt{d}}\right).$$

The factual attention response is obtained under the observed representation:


(13) $$R_{f}=\text{Attn}(q,K,V)=A_{\mathrm{att}}V.$$

Since V contains both lesion-specific local evidence and contextual information, $R_{f}$ may be affected by contextual confounding.

To construct a counterfactual response, we perform a representation-level intervention on the value tokens. Specifically, the local value tokens are replaced by a global contextual prototype:


(14) $$\bar{v}=\frac{1}{N}\sum_{i=1}^{N}v_{i},$$

(15) $$V_{cf}=[\bar{v},\bar{v},\ldots ,\bar{v}]\in R^{N\times d}.$$

This operation can be interpreted as the intervention


(16) $$do(V=V_{cf}),$$

which removes local spatial variations from the value branch while preserving a homogeneous global contextual prototype. The query q and key matrix K are kept unchanged, so the factual and counterfactual responses are compared under the same attention assignment. The counterfactual response is then computed as


(17) $$R_{cf}=\text{Attn}(q,K,do(V=V_{cf}))=A_{\mathrm{att}}V_{cf}.$$

The lesion-specific causal effect is estimated by subtracting the counterfactual context-only response from the factual response:


(18) $$E_{c}=R_{f}-R_{cf}=A_{\mathrm{att}}(V-V_{cf}).$$

This contrast corresponds to a representation-level controlled direct effect under fixed query and key conditions. It estimates the portion of the attention response that cannot be explained by homogeneous contextual information. In other words, if a response is mainly caused by global context, it will appear in both $R_{f}$ and $R_{cf}$ and will be suppressed by subtraction. Conversely, lesion-discriminative local variations are preserved in $E_{c}$. We emphasize that this is not intended to estimate a population-level clinical causal effect, but rather a model-internal causal contrast for reducing contextual dependency in weakly supervised representation learning.

#### 2.1.3 Orthogonal causal regularization

Although the counterfactual intervention suppresses context-dominated responses, residual feature overlap may still exist between the estimated causal effect representation and the contextual response. To further encourage disentanglement, we introduce an orthogonal regularization term based on cosine similarity:


(19) $$L_{\text{orth}}=\frac{1}{B}\sum_{b=1}^{B}\left|\frac{E_{c}^{\left(b\right)}\cdot R_{cf}^{\left(b\right)}}{{\left\|E_{c}^{\left(b\right)}\right\|}_{2}{\left\|R_{cf}^{\left(b\right)}\right\|}_{2}+\epsilon }\right|,$$

where *B* denotes the batch size, $||\cdot |{|}_{2}$ is the Euclidean norm, and ϵ is a small constant used for numerical stability. Minimizing $L_{\text{orth}}$ encourages the lesion-specific causal effect representation $E_{c}$ and the contextual response $R_{cf}$ to occupy complementary feature subspaces, thereby reducing information leakage between the two pathways.

#### 2.1.4 Causal feature reweighting

The estimated causal effect vector is transformed into a channel-wise modulation signal:


(20) $$G=\sigma (W_{g}E_{c}+b_{g}),$$

where $W_{g}$ and $b_{g}$ are learnable parameters and σ(·) denotes the Sigmoid activation function. The gating signal is used to recalibrate patch representations:


(21) $$\tilde{X}=X+G⊙X,$$

where ⊙ denotes element-wise multiplication. Through this residual gating mechanism, channels associated with lesion-specific causal evidence are enhanced, while context-dominated channels are suppressed. The resulting causal-refined representation $\tilde{X}$ is used as the input to the subsequent graph reasoning stage.

### 2.2 Multi-scale spectral graph reasoner

Although the proposed CCP module mitigates contextual confounding and enhances lesion-specific representations, histopathology images exhibit complex multi-scale spatial dependencies that cannot be fully captured by independent patch features. These dependencies range from local cellular structures to global tissue architectures, which are essential for accurate weakly supervised localization.

To address this, we propose a *Multi-scale Spectral Graph Reasoner (MSGR)* that models patch relationships via topology-aware spectral propagation. Unlike conventional GNNs that rely on iterative neighborhood aggregation and are prone to over-smoothing, MSGR performs multi-scale spectral filtering using Chebyshev polynomial approximation, enabling effective long-range dependency modeling while preserving structural boundaries.

#### 2.2.1 Multi-scale topology construction

Given the causal-refined patch representation


(22) $$\tilde{X}=[{\tilde{x}}_{1},{\tilde{x}}_{2},\ldots ,{\tilde{x}}_{N}]\in R^{N\times d},$$

each patch token is treated as a graph node and arranged according to its original spatial position on the image grid. Let


(23) $$G^{\left(s\right)}=(V,E^{\left(s\right)})$$

denote the graph constructed at the *s*-th topology scale, where $V={\left\{v_{i}\right\}}_{i=1}^{N}$ is the node set, $E^{\left(s\right)}$ is the edge set, and s=1,…,S indexes the topology scale. The adjacency matrix of the *s*-th graph is defined as


(24) $$A^{\left(s\right)}\in R^{N\times N},$$

(25) $$A_{ij}^{\left(s\right)}=\{\begin{array}{cc} 1, & v_{j}\in N^{\left(s\right)}(v_{i}),\\ 0, & \text{otherwise}, \end{array}$$

where $N^{\left(s\right)}(v_{i})$ denotes the predefined spatial neighborhood of node $v_{i}$ at scale *s*. Smaller neighborhoods encode local tissue continuity, whereas larger neighborhoods capture long-range tissue dependencies.

Self-loops are added to preserve node identity information:


(26) $${\tilde{A}}^{\left(s\right)}=A^{\left(s\right)}+I_{N},$$

where $I_{N}$ is the N×N identity matrix. The degree matrix is defined as


(27) $$D_{ii}^{\left(s\right)}=\sum_{j=1}^{N}{\tilde{A}}_{ij}^{\left(s\right)}.$$

The row-normalized adjacency matrix is then obtained as


(28) $${\hat{A}}^{\left(s\right)}={\left(D^{\left(s\right)}\right)}^{-1}{\tilde{A}}^{\left(s\right)}.$$

The set ${\left\{{\hat{A}}^{\left(s\right)}\right\}}_{s=1}^{S}$ provides multiple topology views of the same pathology image and enables structural reasoning across different spatial scales.

#### 2.2.2 Spectral chebyshev graph convolution

Given an input feature matrix


(29) $$H\in R^{N\times d},$$

the Chebyshev bases at the *s*-th topology scale are defined as


(30) $$T_{0}^{\left(s\right)}=H,$$

(31) $$T_{1}^{\left(s\right)}={\hat{A}}^{\left(s\right)}H,$$

(32) $$T_{2}^{\left(s\right)}=2{\hat{A}}^{\left(s\right)}T_{1}^{\left(s\right)}-T_{0}^{\left(s\right)}.$$

Here, $T_{0}^{\left(s\right)}$ preserves the original node information, $T_{1}^{\left(s\right)}$ aggregates direct neighborhood information, and $T_{2}^{\left(s\right)}$ introduces second-order spectral responses that help preserve high-frequency structural variations such as tissue boundaries.

For the *s*-th topology scale, the spectral response is computed as


(33) $$H^{\left(s\right)}=T_{0}^{\left(s\right)}W_{0}^{\left(s\right)}+T_{1}^{\left(s\right)}W_{1}^{\left(s\right)}+T_{2}^{\left(s\right)}W_{2}^{\left(s\right)},$$

where


(34) $$W_{0}^{\left(s\right)},W_{1}^{\left(s\right)},W_{2}^{\left(s\right)}\in R^{d\times d}$$

are learnable transformation matrices for different Chebyshev orders. The outputs from all topology scales are aggregated with a self-projection pathway:


(35) $$H^{\prime }=HW_{\text{self}}+\sum_{s=1}^{S}H^{\left(s\right)},$$

where $W_{\text{self}}\in R^{d\times d}$ preserves node-specific identity information. Finally, the SCGC output is obtained using a residual connection:


(36) $$\text{SCGC}(H)=H+H^{\prime }.$$

The residual formulation facilitates optimization and helps alleviate over-smoothing during deep graph propagation.

#### 2.2.3 Deep multi-scale graph reasoning

A single graph convolution layer can only capture limited structural dependencies. To model complex tissue organization, multiple SCGC layers are sequentially stacked.

Let


(37) $$H^{\left(0\right)}=\tilde{X}$$

denote the CCP-enhanced patch representation.

The graph propagation process is recursively defined as


(38) $$H^{\left(l+1\right)}=\text{SCGC}\left(H^{\left(l\right)}\right),\;l=0,\ldots ,L-1,$$

where *L* denotes the graph reasoning depth.

Through repeated multi-scale spectral propagation, each node progressively incorporates structural information from increasingly larger spatial regions while retaining local boundary details.

The final graph representation is obtained through layer normalization:


(39) $$H_{\mathrm{graph}}=\text{LayerNorm}\left(H^{\left(L\right)}\right).$$

The resulting feature embeddings simultaneously encode lesion-specific causal evidence and topology-aware structural information, providing a robust representation for downstream segmentation prediction.

### 2.3 Segmentation prediction and joint optimization

The graph-enhanced representation $H_{\text{graph}}$ is fed into a transformer-based decoder to generate dense semantic predictions:


(40) $$M_{\text{patch}}=\text{Decoder}(H_{\text{graph}}),$$

where $M_{\text{patch}}\in R^{N\times C}$ and *C* denotes the number of tissue categories. Bilinear interpolation is applied to recover pixel-level segmentation maps:


(41) $$M=\text{Interp}(M_{\text{patch}}),\;M\in R^{H\times W\times C}.$$

The overall training objective consists of the weakly supervised segmentation loss and the orthogonal causal regularization term:


(42) $$L=L_{\text{seg}}+\lambda L_{\text{orth}},$$

where $L_{\text{seg}}$ denotes the weakly supervised segmentation objective, $L_{\text{orth}}$ is the causal orthogonal regularization term, and λ≥0 is a balancing coefficient controlling the strength of causal disentanglement. By jointly optimizing these objectives, CSS learns lesion-discriminative representations, suppresses contextual confounding, and preserves fine-grained structural information.

## 3 Experiments

In this section, we comprehensively evaluate the proposed *Causal Spectral Segmenter (CSS)* on two publicly available weakly supervised histopathology segmentation benchmarks. We aim to validate the effectiveness of the proposed causal counterfactual learning strategy and multi-scale spectral graph reasoning module under challenging real-world pathology scenarios.

We first introduce the datasets and implementation details, followed by comparisons with state-of-the-art weakly supervised segmentation methods. Finally, we report quantitative results using standard evaluation metrics.

### 3.1 Datasets

We conduct experiments on two weakly supervised histopathology segmentation benchmarks, namely LUAD-HistoSeg and BCSS-WSSS, covering lung and breast cancer tissues, respectively. LUAD-HistoSeg contains lung adenocarcinoma (LUAD) patches with image-level annotations Han *et al.* (2022), spanning four tissue categories: Tumor Epithelial (TE), Tumor-Associated Stroma (TAS), Lymphocyte (LYM), and Necrosis (NEC). It consists of 17 285 patches of size 224×224, including 16 678 training samples with image-level labels and 607 samples (300 validation and 307 test) with pixel-level annotations for evaluation. BCSS-WSSS is derived from breast cancer H&E-stained whole-slide images Han *et al.* (2022), and includes four categories: Tumor (TUM), Stroma (STR), Lymphocytic infiltrate (LYM), and Necrosis (NEC). The dataset contains 31 826 patches of size 224×224, split into 23 422 training, 3418 validation, and 4986 test samples, with pixel-level annotations provided for validation and testing. Both datasets exhibit strong inter-class ambiguity and intra-class variability across different cancer types, making them suitable benchmarks for weakly supervised segmentation.

### 3.2 Implementation details

All experiments are implemented in PyTorch and conducted on a single NVIDIA RTX 3090Ti GPU. Input images are resized to 224×224 and divided into non-overlapping patches with a patch size of P=16. We adopt ViT-S/16 as the backbone encoder with an embedding dimension of D=384 and 12 transformer layers. For the proposed CSS framework, the multi-scale spectral graph reasoning module consists of L=8 stacked layers. The graph topology is constructed using multiple spatial connectivities (e.g. {4,8,12,16}) to capture both local and long-range tissue dependencies. A transformer-based mask decoder is employed to generate dense segmentation predictions from graph-enhanced patch representations. The model is optimized using stochastic gradient descent (SGD) with an initial learning rate of $1\times 10^{-2}$. The batch size is set to 32, and the total number of training epochs is 10.

### 3.3 Comparison methods

We compare the proposed CSS with representative weakly supervised histopathology segmentation methods, including CNN-based, pseudo-label-based, synthesis-based, and vision-language-driven approaches. HistoSegNet Chan *et al.* (2019) is a CNN-based framework that learns region localization from image-level supervision. OEEM Li *et al.* (2022) improves weak supervision via online example mining for reliable sample selection. WSSS-Tissue Han *et al.* (2022) adopts a two-stage pseudo-labeling strategy with refinement. TPRO Zhang *et al.* (2023) incorporates text prompts to enhance pseudo mask quality using language priors. PistoSeg Fang *et al.* (2023) reformulates weak supervision into a fully supervised setting through synthesis and consistency learning. HisynSeg Fang *et al.* (2025) integrates image synthesis with self-supervised learning to improve robustness. PLIMSeg Ding *et al.* (2026) leverages vision-language alignment for improved localization.

For a fair and transparent comparison, the reported results of the baseline methods are directly adopted from their original papers under the corresponding dataset protocols. We do not re-implement these baselines. The results of CSS are obtained using our implementation under the same dataset splits and evaluation protocol.

### 3.4 Evaluation metrics

We use per-category Intersection over Union (IoU) and mean Intersection over Union (mIoU) to evaluate segmentation performance. It should be noted that the table columns such as TE, TAS, LYM, NEC, TUM, and STR denote tissue categories, not evaluation metrics. The value reported under each tissue category is the IoU score of that category.

For a tissue category *c*, IoU is defined as


(43) $${\text{IoU}}_{c}=\frac{\left|{\hat{M}}_{c}\cap M_{c}\right|}{\left|{\hat{M}}_{c}\cup M_{c}\right|},$$

where ${\hat{M}}_{c}$ and $M_{c}$ denote the predicted and ground-truth pixel sets of category *c*, respectively. IoU measures the spatial overlap between prediction and ground truth. A higher IoU indicates more accurate localization and boundary delineation for the corresponding tissue category.

The mean IoU is computed by averaging IoU over all tissue categories:


(44) $$\text{mIoU}=\frac{1}{C}\sum_{c=1}^{C}{\text{IoU}}_{c},$$

where *C* is the number of tissue categories. mIoU reflects the overall segmentation performance across all categories and is less biased toward dominant tissue classes than pixel accuracy. Therefore, per-category IoU evaluates class-specific segmentation quality, while mIoU summarizes the balanced performance of the model across different tissue structures.

### 3.5 Experiment results and analysis

#### 3.5.1 Comparison between CSS and previous works

Table 1 presents the quantitative results on LUAD-HistoSeg and BCSS-WSSS. CSS achieves the best overall performance on both datasets, reaching mIoU scores of *76.91%* and *71.72%*, respectively, exceeding the previous state-of-the-art method PLIMSeg by 0.53% and 0.65%.

**Table 1:** Comparison between CSS and previous works on LUAD-HistoSeg and BCSS-WSSS.

Method	LUAD-HistoSeg					BCSS-WSSS				
	TE	NEC	LYM	TAS	mIoU	TUM	STR	LYM	NEC	mIoU
HistoSegNet	45.59	36.30	58.28	50.81	47.75	33.14	46.46	29.05	1.91	27.64
WSSS-Tissue	74.54	64.48	52.54	58.67	62.55	73.81	68.28	55.38	58.34	64.08
OEEM	73.81	70.49	71.89	69.48	71.42	74.86	64.68	48.91	61.03	62.37
TPRO	74.80	78.20	71.10	69.80	73.50	76.20	65.10	54.20	62.80	64.60
PistoSeg	77.15	78.50	70.83	70.02	74.12	81.16	73.98	56.26	66.73	69.53
HisynSeg	78.23	78.46	77.15	71.19	76.25	81.64	74.67	60.15	66.48	70.73
PLIMSeg	78.72	81.10	73.54	72.14	76.38	81.39	75.41	60.33	67.16	71.07
CSS	78.48	80.63	75.21	73.30	**76.91**	78.95	73.45	65.43	69.05	**71.72**

The columns TE, NEC, LYM, TAS, TUM, and STR denote tissue categories, and the reported values are per-category IoU scores. Baseline results are directly adopted from the corresponding original papers, while CSS results are obtained by our implementation. Specifically, bold values indicate the best performance among all compared methods.

Notably, CSS obtains the best performance on TAS in LUAD-HistoSeg and achieves substantial gains on LYM and NEC in BCSS-WSSS, demonstrating its superior capability in segmenting complex pathological structures. These results validate the effectiveness of integrating causal representation learning with spectral graph reasoning for weakly supervised pathology segmentation.

#### 3.5.2 Ablation study

Table 2 presents the ablation results of CSS. The full model achieves the best performance on both datasets, obtaining mIoU scores of *76.91%* and *71.72%*. Removing MSGR results in the largest performance drop, while excluding CCP also consistently degrades segmentation accuracy, demonstrating the effectiveness of spectral graph reasoning and causal counterfactual learning, respectively. When both modules are removed, the performance decreases further, indicating that CCP and MSGR provide complementary benefits and jointly contribute to the superior performance of CSS.

**Table 2:** Ablation study on LUAD-HistoSeg and BCSS-WSSS.

Method	LUAD-HistoSeg					BCSS-WSSS				
	TE	NEC	LYM	TAS	mIoU	TUM	STR	LYM	NEC	mIoU
CSS	78.48	80.63	75.21	73.30	**76.91**	78.95	73.45	65.43	69.05	**71.72**
without MSGR	76.85	77.3	70.41	71.74	74.07	79.71	72.88	56.05	65.45	68.52
without CCP	77.6	81.08	73.72	72.73	76.28	79.13	73.19	61.13	68.23	70.42
without MSGR & CCP	76.6	76.45	70.13	79.16	73.34	79.92	73.21	57.20	69.45	69.95

The columns TE, NEC, LYM, TAS, TUM, and STR denote tissue categories, and the reported values are per-category IoU scores. Baseline results are directly adopted from the corresponding original papers, while CSS results are obtained by our implementation.

#### 3.5.3 Effect of graph reasoning depth

Table 3 reports the impact of graph reasoning depth *l*. Performance consistently improves as *l* increases from 6 to 8, reaching the highest mIoU of *76.91%* on LUAD-HistoSeg and *71.72%* on BCSS-WSSS. However, further increasing the depth results in a gradual decline, indicating that excessive graph propagation may cause feature over-smoothing. Therefore, l=8 achieves the best trade-off between long-range dependency modeling and boundary preservation.

**Table 3:** Effect of graph reasoning depth *l* on LUAD-HistoSeg and BCSS-WSSS.

CSS	LUAD-HistoSeg					BCSS-WSSS				
	TE	NEC	LYM	TAS	mIoU	TUM	STR	LYM	NEC	mIoU
*l *= 6	76.75	78.46	72.69	71.47	74.84	80.44	73.93	60.05	68.29	70.68
*l *= 7	78.64	79.20	73.59	73.02	76.11	79.28	73.63	61.42	67.61	70.48
*l *= 8	78.48	80.63	75.21	73.30	**76.91**	78.95	73.45	65.43	69.05	**71.72**
*l *= 9	77.92	78.88	73.59	72.54	75.73	80.98	74.27	60.93	68.18	71.09
*l *= 10	76.64	78.27	71.66	71.62	74.51	79.45	73.21	62.68	65.45	70.20

The columns TE, NEC, LYM, TAS, TUM, and STR denote tissue categories, and the reported values are per-category IoU scores. Baseline results are directly adopted from the corresponding original papers, while CSS results are obtained by our implementation.

#### 3.5.4 Effect of multi-scale connectivity on graph reasoning

Table 4 studies multi-scale connectivity configurations. Results show that using multiple connectivity scales consistently improves performance over limited or overly dense graphs. The best performance is achieved with (6,8,10,12,14) on LUAD-HistoSeg (*76.91%*) and (10,12,14) on BCSS-WSSS (*71.72%*). In contrast, overly large neighborhoods degrade performance due to redundant context and over-smoothing, indicating the importance of a well-balanced multi-scale graph design.

**Table 4:** Effect of different multi-scale connectivity configurations on LUAD-HistoSeg and BCSS-WSSS.

CSS	LUAD-HistoSeg					BCSS-WSSS				
*l *= 8	TE	NEC	LYM	TAS	mIoU	TUM	STR	LYM	NEC	mIoU
(10,12)	77.18	78.18	74.65	72.46	75.62	80.25	74.24	61.40	66.61	70.62
(12,14)	78.37	78.99	74.79	72.57	76.18	79.07	73.14	61.05	68.88	70.54
(1,01,214)	77.76	77.36	70.39	69.81	73.83	78.95	73.45	65.43	69.05	**71.72**
(1,21,416)	77.48	77.27	73.10	72.08	74.98	78.24	72.94	60.73	67.26	69.79
(81,01,214)	76.51	75.63	73.59	72.43	74.54	79.67	73.63	62.23	67.68	70.80
(1,01,21,416)	75.87	74.12	72.33	69.61	72.98	79.74	73.55	61.26	66.13	70.17
(81,01,21,416)	77.48	77.84	73.63	72.94	75.47	78.81	73.38	61.15	68.68	70.51
(6,81,01,214)	78.48	80.63	75.21	73.30	**76.91**	80.22	72.98	60.10	67.23	70.13
(6,81,01,21,416)	77.78	81.55	73.92	73.11	76.59	78.26	72.83	57.94	68.55	69.39

The columns TE, NEC, LYM, TAS, TUM, and STR denote tissue categories, and the reported values are per-category IoU scores. Baseline results are directly adopted from the corresponding original papers, while CSS results are obtained by our implementation. Specifically, bold values indicate the best performance among all compared methods.

#### 3.5.5 Qualitative interpretation of CCP

**Figure 2:**
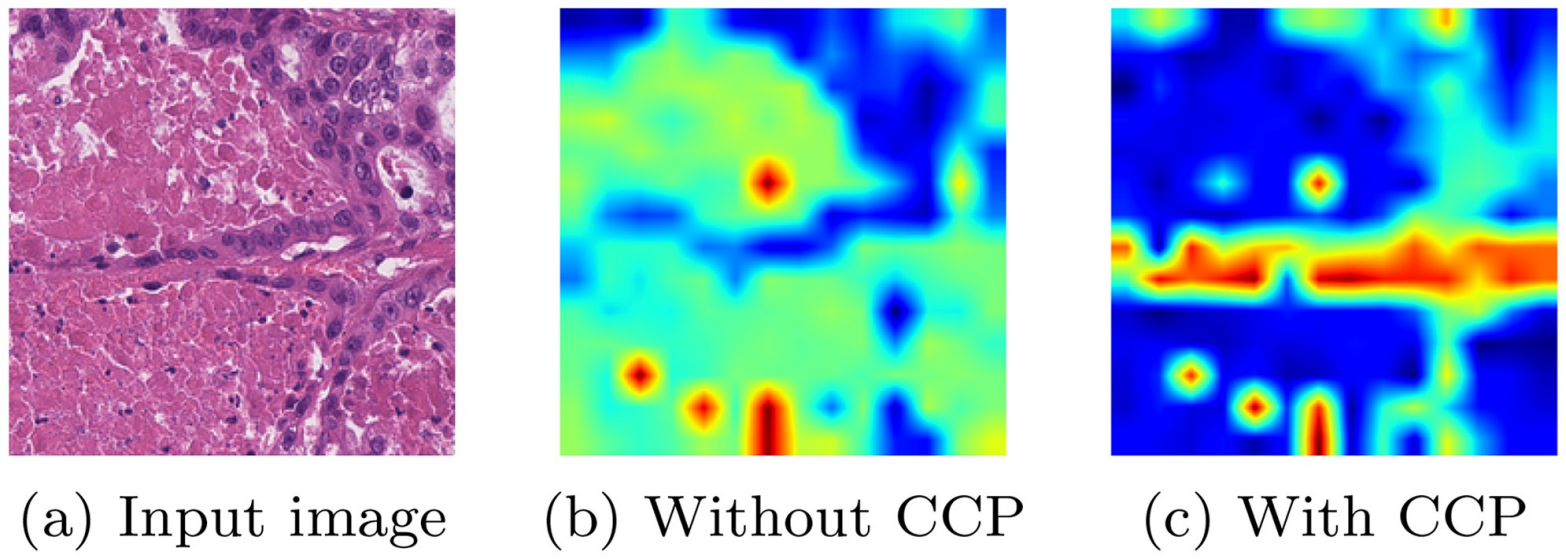
Heatmap on LUAD-HistoSeg. Qualitative comparison of activation maps before and after applying CCP. From left to right: input image, activation map without CCP, activation map with CCP. Compared with the baseline, CCP suppresses irrelevant contextual responses and produces more lesion-focused localization.

To further verify whether the proposed CCP reduces contextual dependency, we provide qualitative visualizations of activation maps before and after applying CCP. As shown in Fig. 2, the baseline model without CCP often produces dispersed responses over contextual regions, such as surrounding stroma, background tissue, or visually correlated non-lesion areas. In contrast, CSS with CCP generates more compact and lesion-focused activation maps. The highlighted regions are better aligned with the ground-truth tissue regions, and irrelevant contextual responses are visibly suppressed.

These observations indicate that the improvement brought by CCP is not merely due to increased representation capacity. Instead, CCP changes the spatial attention behavior of the model by reducing reliance on context-dominated cues and enhancing lesion-specific evidence. This supports the causal motivation of the proposed factual–counterfactual intervention and provides intuitive evidence for improved weakly supervised lesion localization.

## 4 Conclusion

We proposed a Causal Spectral Segmenter (CSS) for weakly supervised pathology segmentation. CSS jointly addresses contextual confounding and graph over-smoothing by integrating a Causal Counterfactual Projector (CCP) and a Multi-scale Spectral Graph Reasoner (MSGR). The CCP module introduces a representation-level factual–counterfactual intervention to estimate lesion-specific causal effects and suppress context-dominated responses, while MSGR performs multi-scale spectral topology reasoning to model tissue structures and preserve lesion boundaries.

Extensive experiments on LUAD-HistoSeg and BCSS-WSSS demonstrate that CSS consistently outperforms existing weakly supervised pathology segmentation methods. Additional qualitative activation-map visualizations further show that CCP reduces reliance on irrelevant contextual regions and improves lesion-focused localization. These results validate the effectiveness of combining causal representation learning with spectral graph modeling for robust weakly supervised pathology segmentation.
